# Evaluation of the effect of maternally derived antibody on response to MMR vaccine in Thai infants

**DOI:** 10.1016/j.vaccine.2022.01.049

**Published:** 2022-03-01

**Authors:** Siyuan Hu, Nicola Logan, Jiratchaya Puenpa, Nasamon Wanlapakorn, Sompong Vongpunsawad, Yong Poovorawan, Brian J. Willett, Margaret J. Hosie

**Affiliations:** a MRC-University of Glasgow Centre for Virus Research, Bearsden Road, Glasgow G61 1QH, UK; b Center of Excellence in Clinical Virology, Department of Pediatrics, Faculty of Medicine, Chulalongkorn University, Bangkok, Thailand

**Keywords:** Childhood vaccination, Measles-mumps-rubella (MMR), Maternal immunity, Neutralising antibody, Pseudotype-based virus neutralisation assay

## Abstract

**Background:**

Although the number of measles cases declined globally in response to anti-measles immunisation campaigns, measles has re-emerged. A review of current vaccination policies is required to improve measles elimination strategies.

**Methods:**

A pseudotype-based virus neutralisation assay (PVNA) was used to measure neutralising antibody titres in serum samples collected from Thai infants at six timepoints before and after two-doses of MMR (1&2) vaccination (ClinicalTrials.gov no. NCT02408926). Vesicular stomatitis virus (VSV) luciferase pseudotypes bearing the haemaglutinin (H) and fusion (F) glycoproteins of measles virus (MeV) were prepared. Serial dilutions of serum samples were incubated with VSV (MeV) pseudotypes and plated onto HEK293-human SLAM1 cells; the neutralising antibody titre was defined as the dilution resulting in 90% reduction in luciferase activity.

**Results:**

Neutralising antibody titres in infants born with high levels of maternal immunity (H group) persisted at the time of the first MMR vaccination, and those infants did not respond effectively by developing protective titres. In contrast, infants with lower maternal immunity (L group) developed protective titres of antibody following vaccination. Responses to the second MMR vaccination were significantly higher (P = 0.0171, Wilcoxon signed-rank test) in the H group. The observed correlation between anti-MeV IgG level and neutralising antibody titre in Thai infants indicates the possibility of using rapid IgG testing as a surrogate measure for neutralising activity to define clinical protection levels within populations.

**Conclusion:**

These results demonstrate that varying the timing of the first MMR immunisation according to the level of acquired maternal immunity could increase vaccination immunogenicity and hence accelerate measles eradication.

## 1 Introduction

Measles is one of the most contagious respiratory diseases. Measles virus (MeV) causes significant mortality amongst children, which makes it a priority for public health. Fortunately, measles is a vaccine-preventable disease, and so elimination could be achieved by high, global vaccination coverage. Following the worldwide anti-measles immunisation campaign, the number of annual cases globally declined by 75% and the number of deaths decreased by 79% between 2000 and 2015 [1]. However, measles has re-emerged in recent years around the world, as a result of various factors including waning immunity [2], inadequate vaccine coverage [3], MeV genetic diversity and international importation [4], [5]. Due to differences between the levels of population-wide immunity, local epidemiological situations and the strength of national public health systems, it is important to monitor the immunogenicity of current vaccination policies. More specific improvements might be required to optimise measles vaccination protocols in different geographic regions and population groups.

The measles vaccine was introduced in Thailand in 1984 and a 2nd dose of the vaccine has been administered to 6 year-old children since 1996. The trivalent combined measles-mumps-rubella (MMR) vaccine has been used since its introduction in 1997 [6]. Subsequently, in response to sporadic measles outbreaks that occurred countrywide, Thailand revised the vaccination schedule so that the second dose of MMR vaccine (MMR2) was administered to 2.5 year-old children, rather than 6-year-olds in 2014. The first dose (MMR1) was still administered at 9 months of age. Vaccination coverage for the first dose (MMR1) was 83%-92%, while the rate for MMR2 increased from 3.3% to 90% during the period between 2014 and 2019 [7], [8]. Despite the continuous vaccination of children, the numbers of annual measles cases in Thailand increased sharply and the occurrence of symptoms in children and even infants called into question the immunogenicity of the measles immunisation policy that had been revised in 2014 [9]. So far, the only study that investigated the immunogenicity of the revised policy examined the levels of anti-measles IgG [8].

The measurement of IgG is used for routine clinical analysis as the test is easy to apply; however, enzyme-linked immunosorbent assays (ELISA) are routinely based on either recombinant nucleoprotein or inactivated whole virus and hence may not detect antibodies recognising epitopes on the viral surface glycoproteins that are associated with clinical protection [10], [11]. Neutralising antibodies bind directly to the surface glycoproteins haemagglutinin (H) and fusion (F) that are responsible for receptor attachment and host cell entry of MeV [12], [13]. The neutralising responses of sera from infants were measured in this study to investigate potential factors contributing to vaccine failure, such as MeV genotype diversity or maternal immunity.

Neutralising antibody responses against MeV were measured in serum samples collected from infants who had been vaccinated with two doses of MMR vaccine at 9 months and 2.5 years of age. Samples were collected from 6 timepoints (0, 2, 7, 18, 24 and 36 months of age) from the same cohort that was recruited for a previous study examining IgG responses [8]. The cohort had originally been established as part of a pertussis vaccination programme and the mothers of the infants had received the Tdap vaccine during pregnancy [14]. In this study the titres of measles neutralising antibodies were determined using a pseudotype-based virus neutralisation assay (PVNA) to measure activity against the vaccine strain (Edmonston) and the two major genotypes (B3 and D8) that are circulating in Thailand [6]. The aim of the study was to examine the immunogenicity of the measles immunisation policy in Thailand that was revised in 2014. The responses induced by vaccination and the potential to cross-neutralise the B3 and D8 MeV genotypes were evaluated. The results demonstrated that the current vaccination programme induced strong neutralising antibody responses in infants with low levels of maternal immunity and provided good cross-neutralising antibody responses against diverse genotypes. However, infants in the cohort who had acquired high levels of maternal immunity failed to respond to the MMR1 vaccine but responded to MMR2. Therefore, it was concluded that postponing vaccination in the group with high levels of maternal immunity would likely lead to more effective immunisation of these infants.

## 2 Materials and methods

### 2.1 Serum samples and cell lines

Serum samples of infants comprised part of a larger cohort originally established for a study examining responses to pertussis vaccination in Thailand; the pertussis study (ClinicalTrials.gov NCT02408926) was approved by the Institutional Review Board at Chulalongkorn University and the Ethical Committee of the University of Antwerp. The pregnant mothers had previously been vaccinated with tetanus-diphtheria-acellular pertussis (Tdap) vaccine (Boostrix; GlaxoSmithKline Biologicals, Rixensart, Belgium) during the third trimester. The infants were vaccinated with MMR vaccine (Priorix; GlaxoSmithKline Biologicals, Rixensart, Belgium) or M-M-RII (Merck & Co., Kenilworth, NJ) at the ages of 9 months (MMR1) and 2.5 years (MMR2) according to the immunisation policy that was revised in 2014. Other routine or optional vaccines administered for children, such as bacilli Calmette-Guerin (BCG) vaccine, inactivated poliovirus (IPV) vaccine and hepatitis A vaccine were detailed previously [14]. Serum samples were collected at six timepoints (0, 2, 7, 18, 24 and 36 months of age) from the infants and tested for measles neutralising antibodies.

The WHO 3rd International Standard Biological Reference human anti-measles serum 97/648 was obtained from NIBSC [15] and was diluted to 120 mIU/mL. The WHO gold standard plaque reduction neutralisation test defines 120 mIU/mL as the threshold above which neutralising antibody levels protect against infection [16].

HEK 293 T [17] and HEK 293 [18] that had been engineered to express human signalling lymphocyte activation molecule (SLAM1) were maintained in Dulbecco’s Modified Eagle Medium (DMEM) supplemented with 10% foetal bovine serum, 100 IU/mL penicillin – streptomycin and 2 mM glutamine. Media for HEK293 cells were supplemented with 1 μg/mL puromycin and media for HEK293T cells were supplemented with 400 μg/mL G418. All media and supplements were obtained from Thermo Fisher Scientific Ltd., Paisley, UK.

### 2.2 Production of vesicular stomatitis virus (measles virus) pseudotypes (VSV (MeV))

VSV (MeV) pseudotypes were prepared from three strains of MeV, namely Edmonston, B3 (MVi/Rotterdam.NLD/32.12/1) and D8 (MVi/Dodewaard.NLD/29.13), kindly provided by Dr. R. de Swart, EMC, Rotterdam. The H genes were amplified and used to prepare VSV (MeV) pseudotypes using the method described previously [19].

### 2.3 Pseudotype-based virus neutralisation assay

Serum dilutions were prepared in triplicate in 96 well culture plates (Greiner Bio-one No. 655083). Each serum sample was diluted in complete DMEM at dilutions of 1:32, 1:64, 1:256, 1:1024, 1:4096, 1:16384 and 1:65536. A volume of 25 μL of each serum dilution was added per well; 25 μL of complete DMEM were added to the “no serum control” wells. Diluted serum samples were incubated with the same volume of titrated and diluted VSV(MeV) pseudotype at 37 °C in an atmosphere of 5% CO_2_ for 40 min before 2x10^4^ HEK293-human SLAM1 cells were added per well. Following incubation at 37 °C in an atmosphere of 5% CO_2_ for 48 h, luciferase substrate (SteadyLite plus^TM^, Pelkin Elmer) was added to each well and the luciferase activity was measured using an EnSight™ multi-mode plate reader (Pelkin Elmer). Antibody titres were calculated by interpolating the point at which there was a 90% reduction in luciferase activity (90% neutralisation, inhibitory concentration 90 or IC_90_) [20].

### 2.4 Enzyme-linked immunosorbent assays (ELISA)

The measles specific IgG titres in sera were measured using EUROIMMUN® ELISA kits at all timepoints. Antibody levels below the lower limit of quantification (50 IU/L) observed in some samples were assigned the arbitrary value of 25 IU/L.

### 2.5 Statistical analysis

Neutralising antibody titres were measured at each timepoint for each infant against different MeV genotypes and were non-normally distributed, hence, titres were compared statistically in pairs using the Wilcoxon matched-pairs signed-rank test to determine whether differences were statistically significant [21].

An unpaired comparison of the neutralising antibody titres between infants with different levels of maternal immunity, which were also non-normally distributed, were statistically compared using the Mann-Whitney *U* test [22].

## 3 Results

### 3.1 Measles neutralising antibody titres against measles virus (vaccine strain Edmonston) in serum samples collected from Thai infants

**Fig. 1:**
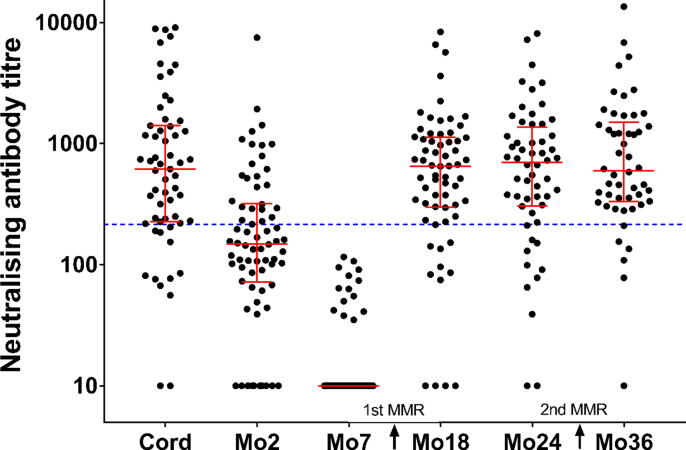
**The distribution of neutralising antibody titres in Thai infants against the Edmonston strain at 6 timepoints.** Median titres with interquartile ranges are shown. The dotted blue line indicates the neutralising antibody titre of the WHO 3rd international standard (NIBSC 97/648) diluted to 120 mIU/mL, which is defined as the clinical protective concentration. (For interpretation of the references to colour in this figure legend, the reader is referred to the web version of this article.)

Neutralising antibody titres against Edmonston were measured in serum samples collected from 70 infants at birth and 2, 7, 18, 24 and 36 months of age (Fig. 1). The infants were immunised with two doses of MMR vaccine at 9 months and 2.5 years (30 months) of age. The median titre decreased to the lowest level at 7 months, which was prior to the first immunisation (MMR1) at 9 months of age that led to an increase in the median titre. The titres were compared with that of the 3rd WHO measles antibody international standard serum (NIBSC 97/648), which was diluted to the defined protective concentration (120 mIU/mL) [16], and the proportion of serum samples with titres greater than or equal to the titre of the standard serum were calculated for each timepoint (Table 1). Protective titres were observed in 79.7% of samples collected at birth (cord blood samples), decreasing to 0% at 7 months and then increasing to 88.0% at 36 months. The proportion of samples with protective titres increased after two doses of MMR vaccine.

**Table 1:** Percentage of infants with neutralising antibody titres greater than the predicted protective threshold.

**Timepoints**	**Percentage of samples (95 %CI)**
**Cord**	79.7 (67.6–88.1)
**Mo2**	37.1 (26.8–48.9)
**Mo7**	0.0 (0.0–7.3)
**Mo18**	81.2 (69.9–89.1)
**Mo24**	80.4 (68.0–88.8)
**Mo36**	88.0 (75.8–94.8)

### 3.2 Infants with lower levels of maternal neutralising antibody against MeV showed robust induction of neutralising antibody after the first MMR vaccination

**Fig. 2:**
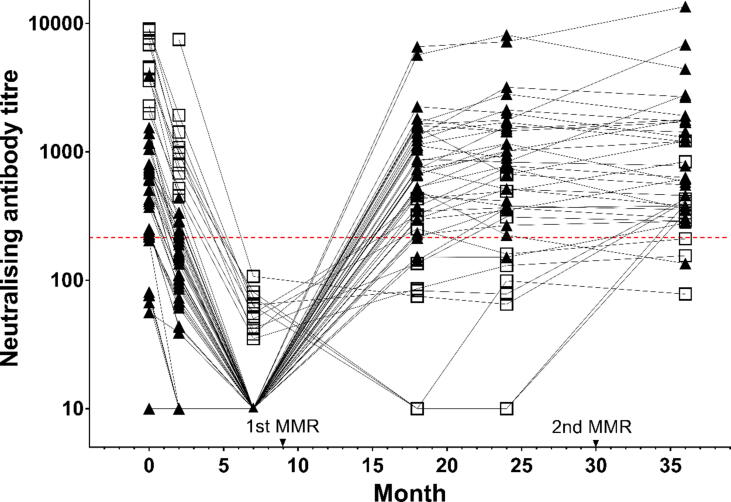
**Neutralising antibody titres of samples collected from Thai infants from birth to 36 months.** The dotted red line indicates the titre of the WHO 3rd international standard (NIBSC 97/648) diluted to 120 mIU/mL, which is defined as the clinical protective concentration. Closed triangles represent 38 of 48 infants who had undetectable neutralising antibody titres at Mo7. The remaining 10 infants with measurable titres at Mo7 are shown as open squares. (For interpretation of the references to colour in this figure legend, the reader is referred to the web version of this article.)

Forty-eight of 70 infants provided serum samples at 4 or more consecutive timepoints. The neutralising antibody titres of these samples were plotted to display the individual responses to measles vaccination (Fig. 2). A range of titres was observed amongst individuals. Infants could clearly be classified into two groups according to their neutralising antibody titres at 7 months; the first group comprising 38 of 48 infants (Fig. 2, filled triangles) displayed no detectable neutralising activity against the vaccine strain Edmonston, while the second group consisting of the remaining 10 infants (Fig. 2, open squares) displayed detectable neutralising activity, albeit below the threshold protective titre. Samples collected at birth (cord blood) from these 10 neutralising antibody-positive infants contained significantly higher titres (P = ****); however, these infants responded poorly (5 of 10 below 120 mIU/mL) to the first measles vaccination at 9 months. In contrast, infants with no detectable neutralising activity at 7 months tended to have acquired lower neutralising antibody titres at birth and displayed stronger neutralising responses following their first vaccination.

**Fig. 3:**
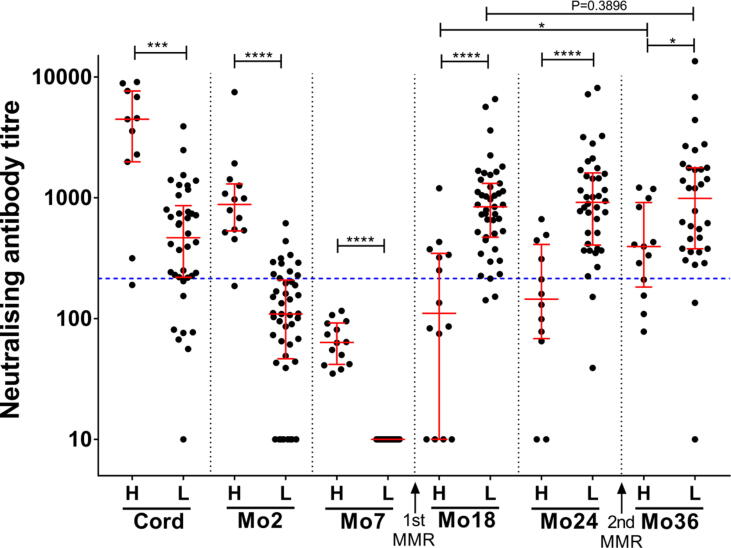
**Comparing neutralising antibody titres between infants with high versus low maternal immunity levels.** Median titres with interquartile ranges are shown. The dotted blue line indicates the neutralising antibody titre of the WHO 3rd international standard (NIBSC 97/648) diluted to 120 mIU/mL, which is defined as the clinical protective concentration. Asterisks indicate statistical significance (**** P values less than 0.0001; *** P values less than 0.001; * P values less than 0.05). Neutralising antibody titres of infants with high maternal immunity (H) were compared to the titres of infants in low maternal immunity group (L) at each timepoint (Mann-Whitney test). Neutralising antibody titres of samples collected after each dose of MMR vaccination were compared between the H and L groups (Wilcoxon matched-pairs signed-rank test). (For interpretation of the references to colour in this figure legend, the reader is referred to the web version of this article.)

Samples at 7 months were available for 59 of the 70 infants recruited for the study, allowing the infants to be classified into two groups based on whether they displayed high (H) or low (L) titres of maternal neutralising antibody at 7 months of age; the titres of these two groups were compared at each timepoint (Fig. 3). Prior to the first measles vaccination, the neutralising activity of samples from infants in group H was significantly higher than that of infants in group L (P = 0.0001 at birth, P < 0.0001 at 2 months and P < 0.0001 at 7 months; Mann-Whitney test). After the first measles vaccination, samples from the infants in group H displayed significantly lower neutralising activity compared to the infants in group L (P < 0.0001 at 18 months, P < 0.0001 at 24 months and P = 0.0107 at 36 months; Mann-Whitney test). The antibody titres of infants within each group were compared between the 18 and 36 months timepoints, to determine the neutralising antibody response to the second vaccination administered at 30 months of age (Fig. 3). Titres were significantly higher after the second dose of vaccine compared to the first dose of vaccine in infants in group H, whereas no significant difference was observed for infants in group L (P = 0.0171 of group H, P = 0.3896 of group L; Wilcoxon signed-rank test).

The titres of the sera in groups H and L were compared with the titre of the WHO 3rd measles antibody international standard serum (NIBSC 97/648) diluted to 120 mIU/mL, defined as the clinical protective threshold. The proportion of samples with titres greater than or equal to the protective threshold was calculated for the two groups at each timepoint (Table 2). In infants in group L with low maternal titres, 79.0% (30 of 38) of samples collected at birth had presumed protective titres, 24.4% (11 of 45) at 2 months and 0% at 7 months. Following the first measles vaccination at 9 months, 95.4% (41 of 43) of the infants in group L had presumed protective titres at 18 months, slightly decreasing to 94.7% (36 of 38) at 24 months and 93.8% (30 of 32) at 36 months. In group H, 90.9% of samples collected at birth and 92.9% collected at 2 months had titres greater than the protective threshold; however, this proportion decreased after measles vaccination to only 42.9% at 18 months and 33.2% at 24 months. Following the second measles immunisation, the proportion of infants with presumed protective titres increased to 69.2% at 36 months. Hence, a strong maternal antibody response can interfere with the immunogenicity of the MMR vaccine and result in decreased protection against measles.

**Table 2:** Percentage of infants with neutralising antibody titres greater than the predicted protective threshold.

**Timepoints**	**Percentage of samples (95 %CI)**	
	**High maternal group**	**Low maternal group**
**Cord**	90.9 (60.1->99.9)	79.0 (63.4–89.2)
**Mo2**	92.9 (66.5->99.9)	24.4 (14.1–38.8)
**Mo7**	0.0 (0.0–25.2)	0.0 (0.0–9.4)
**Mo18**	42.9 (21.3–67.4)	95.4 (83.7–99.6)
**Mo24**	33.3 (13.6–61.2)	94.7 (81.8–99.5)
**Mo36**	69.2 (42.0–87.6)	93.8 (78.8–99.3)

### 3.3 Antibodies that cross-neutralised circulating genotypes of measles virus in serum samples from Thai infants

**Fig. 4:**
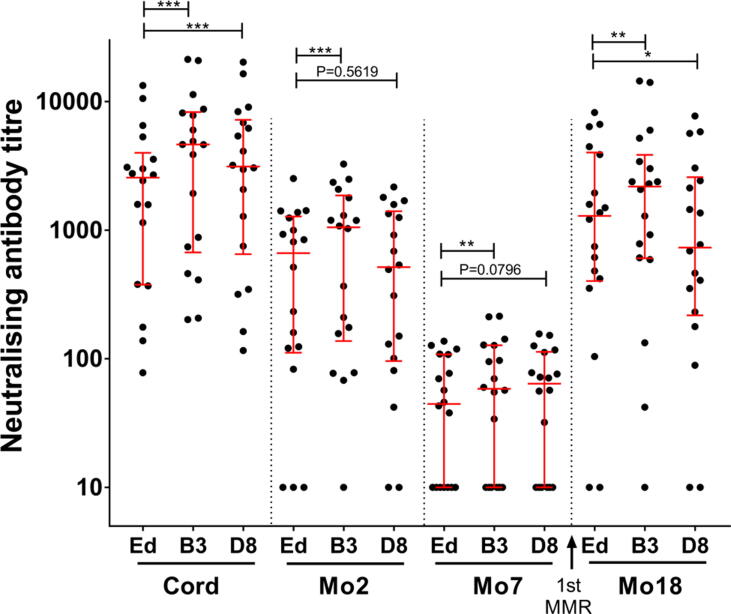
**Cross-neutralising antibodies against circulating measles strains B3 and D8 compared to Edmonston vaccine strain.** Median titres with interquartile ranges are shown and the Wilcoxon signed-rank test was used for statistical analysis. Asterisks indicate statistical significance (*** P values less than 0.001; ** P values less than 0.01; * P values less than 0.05). Neutralising antibody titres against B3 were significantly higher than the titres against Edmonston at all four timepoints tested. Titres against D8 were significantly higher than the titres against Edmonston at birth and were significantly lower at 18 months.

Neutralising activities against the Edmonston vaccine strain, and the circulating strains B3 and D8 were compared in 72 serum samples collected from 18 infants at birth, 2, 7 and 18 months of age (Fig. 4). Titres against the B3 strain were significantly higher compared to the titres against Edmonston strain at all timepoints (P = 0.0002 at birth, P = 0.0003 at 2 months, P = 0.0063 at 7 months, P = 0.0093 at 18 months). Neutralising activity against D8 was significantly higher (P = 0.0001) at birth and lower (P = 0.0290) at 18 months compared to the titres against Edmonston (Wilcoxon signed-rank test).

The proportion of infants with protective titres was calculated for each timepoint by comparing with the 3rd WHO measles antibody international standard serum (NIBSC 97/648) (Table 3). A smaller proportion of infants displayed protective titres against Edmonston at birth and against D8 at 18 months compared to the proportions with protective titres against the other two genotypes tested.

**Table 3:** Percentage of infants with neutralising antibody titres above the protected threshold against different measles virus strains.

**Timepoints**	**Percentage of samples (95 %CI)**		
	**Edmonston**	**B3**	**D8**
**Cord**	83.3 (60.0–95.0)	88.9 (66.0–98.1)	88.9 (66.0–98.1)
**Mo2**	61.1 (38.5–79.8)	61.1 (38.5–79.8)	61.1 (38.5–79.8)
**Mo7**	0.0 (0.0–20.7)	0.0 (0.0–20.7)	0.0 (0.0–20.7)
**Mo18**	83.3 (60.0–95.0)	83.3 (60.0–95.0)	72.2 (48.8–87.8)

### 3.4 Correlation between measles specific IgG levels and neutralising antibody titres

**Fig. 5:**
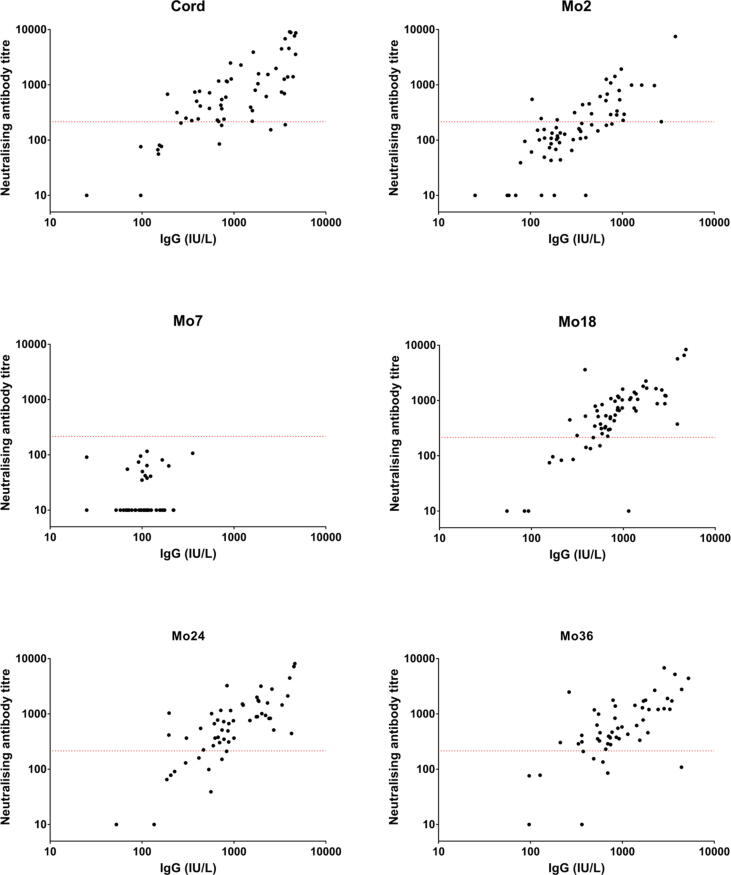
**Correlation between measles specific IgG levels and neutralising antibody titres in Thai infants.** The correlation between measles specific IgG and neutralising antibody titres were calculated (Rs = 0.47 at Cord, 0.79 at Mo2, 0.25 at Mo7, 0.76 at Mo18, 0.74 at Mo24 and 0.68 at Mo36; P value < 0.0001 at Cord, Mo2, Mo18, Mo24 and Mo36, P = 0.0571 at Mo7; Spearman’s rank correlation coefficient). The red lines indicate the titre of the 3rd WHO reference serum at 120 mIU/mL, defined as the protective titre. (For interpretation of the references to colour in this figure legend, the reader is referred to the web version of this article.)

Spearman’s rank correlation coefficients were calculated for IgG and neutralising antibody titres (Fig. 5, Table 4). Sample sets were incomplete for some infants; Fig. 5 displays results from 59 cord samples, 70 samples collected at Mo2, 59 at Mo7, 64 at Mo18, 56 at Mo24 and 55 at Mo36. IgG titres correlated positively with neutralising antibody titres at all timepoints, except for Mo7 when maternal antibody had waned.

**Table 4:** Spearman’s rank correlation coefficient between IgG levels and neutralising antibody titres.

**Timepoints**	**Rs**	**95 %CI**	**P value**
**Cord**	0.74	0.59–0.84	<0.0001
**Mo2**	0.79	0.67–0.86	<0.0001
**Mo7**	0.25	−0.015–0.48	0.0571
**Mo18**	0.76	0.62–0.85	<0.0001
**Mo24**	0.74	0.58–0.84	<0.0001
**Mo36**	0.68	0.5–0.8	<0.0001

## 4 Discussion

### 4.1 Immunogenicity of revised Thai measles vaccination strategy 2014

A major goal of this study was to evaluate the immunogenicity of the current measles vaccination schedule adopted in Thailand, immunising infants at 9 months and 2.5 years of age. It was observed that 81.2% of the samples tested displayed neutralising activity against MeV above the “well-protected” threshold following the first measles immunisation, which is less than the 95% target vaccination coverage proposed by the WHO to achieve herd immunity. However, the proportion of infants with antibody titres above the “well-protected” threshold was 95.4% when samples from infants with higher titres of maternal antibody (group H) were excluded. This finding indicates that it should be possible to boost sufficient titres of neutralising antibody to achieve the measles elimination standard in infants, providing the infants have undetectable titres of neutralising antibody prior to their first measles vaccination. Furthermore, it was shown that neutralising activity induced by measles vaccination effectively cross neutralised genotypes B3 and D8, which are circulating in Thailand [6]. It has been suggested that genetic diversity might contribute to immune escape [23], [24], [25], hence we compared neutralising titres against not only the vaccine strain (Edmonston), but also against representatives of the genotypes circulating currently [26]. Our results suggested that the neutralising response elicited by vaccination effectively cross-neutralise currently circulating genotypes.

When the titres at 18 and 36 months were compared, it was observed that the MMR2 boosted neutralising antibody titres in infants born with higher maternal antibody titres (group H); although their median titre remained significantly lower compared to group L. In contrast, no further boosting in neutralising activity was observed in group L. Hence, this study revealed that the second measles immunisation was most effective in boosting immunity in infants in group H; 88% of the entire cohort (including both groups H and L) developed presumed protective titres after two doses of MMR vaccine. The effect of vaccination appeared to be limited when the infants acquired maternal antibody titres near the presumed protective threshold. This finding is consistent with the results of an earlier study that examined the same cohort, and showed that the second measles immunisation did not lead to an increase in the geometric mean titre of anti-measles IgG [8]. Two doses of measles vaccine are recommended for primary immunisation worldwide, although the schedules vary between different countries and regions depending on local epidemiological situations [27]. Thailand revised the schedule of MMR2 administration from 6 years to 2.5 years old in 2014 in response to the sporadic measles outbreaks observed countrywide. The results of this study confirm that, although MMR2 will protect children who did not develop protective titres following MMR1, infants born with high maternal antibody titres appear to be unable to respond to the MMR1 administered at 9 months of age and are therefore susceptible to infection until after their second immunisation; the current immunisation strategy needs to be improved to induce protective titres earlier in these infants. However, given that the MMR vaccine is trivalent, it would be important to consider the effect of postponing vaccination of infants with high levels of immunity to measles. To avoid any unintended consequences, it would be necessary to ensure that such infants would have sufficient protective immunity against mumps or rubella, so that they would not be susceptible to those infections while their measles vaccination was postponed. It is possible that in some circumstances, it would be necessary to use monovalent vaccines to circumvent this potential scenario.

The serum samples in this study were selected from a larger cohort that was established for a programme examining responses to pertussis vaccination and the mothers of the infants had been vaccinated with the Tdap vaccine during pregnancy. Thus, the variability in responses amongst the samples examined in this study might be less compared to the variability in general population. To address this issue, randomly collected samples should be tested to investigate the immune status of the wider population.

### 4.2 Increase immunogenicity of the first measles immunisation

An effective vaccination strategy is designed to induce protective immunity in infants. The immune system of infants takes months, even years, to become sufficiently mature to protect children against infection with pathogens [28], [29], [30]; consequently, it might be inefficient, or even dangerous, to administer the first dose of measles vaccine too early, before the immune system is sufficiently well-developed. However, vaccination should not be too late as waning maternal immunity would leave infants at risk of infection if exposed to pathogens; several studies have suggested that anti-measles maternal immunity can wane before the age of 9 months [31], [32], consistent with observations in the infants in group L in this study. Moreover, previous studies examining primary vaccine failure concluded that acquired maternal antibody was a key factor for improving the vaccination schedule for measles. It was also suggested that maternal anti-MeV antibodies might block epitopes on the vaccine, thus, the vaccine could fail to induce immunity in infants if administered too early [33]. Long lasting maternal immunity might prompt the postponement of the first dose of measles vaccine [34]. Considering these conclusions from previous studies and the results of this one, postponing MMR1 for infants with high maternal immunity could lead to a significant improvement in the induction of strong immunity in infants.

An important question to address in relation to postponing MMR1 is how best to determine whether infants should be immunised early or later, based on the level of acquired maternal neutralising antibody. A straightforward approach might be to determine a threshold titre such that infants with titres below the threshold should be vaccinated. However, infants are extremely vulnerable, with immature organs and slow blood replenishment; routine blood testing of newborn infants is conducted to screen for genetic conditions using capillary blood sampled via heel lancing. Additional blood sampling is generally only recommended for hospitalised infants and the volume of sampling for clinical care or research purposes is strictly limited [35]. Therefore, the direct measurement of neutralising antibody titres in infants might not be feasible. An alternative might be to measure the neutralising antibody titres in mothers, since a correlation has been described between the mother’s immune status and the amount of transferred antibodies and persistence of maternal antibodies in infants [36], [37]. Pregnant women who had been naturally infected with MeV were shown to transfer higher titres of maternal antibodies to their infants, compared to vaccinated pregnant mothers, and the natural infection-induced maternal immunity persisted longer [38], [39], [40], [41]. These findings led to the recommendation that infants born to vaccinated mothers should be vaccinated against measles earlier [42], [43]. Thus, the measurement of mothers’ neutralising antibody titres could allow the classification of infants into groups suited to different measles vaccination schedules.

The commonly suggested routine tests for pregnant women include urine testing, ultrasound scanning, amniocentesis and serial blood tests for many health status indicators, such as hepatitis and HIV screening, pregnancy-associated serum protein, human chronic gonadotropin and Rhesus status. Overall, blood sampling of pregnant women is more convenient and carries fewer risks than sampling infants. Pregnant mothers could be classified according to their anti-MeV neutralising antibody titres and their offspring could be allocated different vaccination schedules. The immunisation history of mothers should also be taken into consideration. In regions with ongoing transmission in which the infection case number is high, many pregnant mothers are likely to have been naturally infected with MeV and consequently their infants would acquire higher titres of transferred antibodies, such that their first measles vaccination should be postponed. However, regions with high measles transmission rates and low vaccination coverage might lack medical resources and so, if an extra test to measure neutralising antibody titres during pregnancy is unaffordable for the local healthcare system, the classification of infants into groups based on their mothers’ immunisation records could be adopted as an alternative. For instance, infants from vaccinated mothers could be immunised at the age of 7 months while the immunisation of infants of mothers who had been naturally infected could be postponed, until 12 months of age.

The correlation between IgG level and neutralising antibody titre in Thai infants presents the possibility of using rapid IgG testing as a surrogate measure for neutralising antibody titre that defines the clinical protection level within the population. Excluding the data from Mo7 (at which timepoint most samples presented negative neutralising antibody titres), the R values ranged from 0.68 to 0.79 at another five timepoints, with P values indicating high significance (P < 0.0001). However, the accuracy of IgG testing to indicate protection would highly depend on the choice of IgG test kit.

An important prerequisite for ensuring vaccination policies being tailored to the individual is to establish the threshold titre that interferes with vaccination, which would allow the classification described above. The next step could be to measure neutralising antibody titres in pregnant mothers at a specific timepoint as well as the titres at a series of timepoints in their infants; a correlation between the mother’s titre and the period that maternal titres exceed the threshold that interferes with vaccination in infants would provide the evidence required to determine the appropriate timepoints for antibody testing during pregnancy and the vaccination schedule for infants.

## 5 Conclusion

In conclusion, the results of this study indicate that infants with high maternal anti-measles antibodies might fail to efficiently respond to MMR1 at the age of 9 months. As MMR2 did not significantly increase immunity in infants at the age of 2.5 years, optimising the timing of MMR1 according to the level of acquired maternal immunity might be more efficient than administering an extra dose, or replacing the current MMR vaccine with a new vaccine. The MMR vaccine used currently is highly effective and provides good cross-neutralising responses against the most recently circulating MeV genotypes. It is proposed that an additional test for measles neutralising antibody titres during pregnancy would allow the classification of infants according to their level of maternal immunity such that different vaccination schedules could be adopted, inducing protective levels of anti-measles immunity in more infants.

### CRediT authorship contribution statement

**Siyuan Hu:** Methodology, Formal analysis, Investigation, Writing – original draft, Writing – review & editing, Funding acquisition. **Nicola Logan:** Methodology, Investigation, Writing – review & editing. **Jiratchaya Puenpa:** Methodology, Formal analysis, Investigation, Writing – review & editing. **Nasamon Wanlapakorn:** Conceptualization, Investigation, Writing – review & editing, Supervision, Project administration, Funding acquisition. **Sompong Vongpunsawad:** Conceptualization, Writing – review & editing, Supervision, Project administration, Funding acquisition. **Yong Poovorawan:** Conceptualization, Writing – review & editing, Supervision, Funding acquisition. **Brian J. Willett:** Conceptualization, Formal analysis, Writing – review & editing, Supervision, Funding acquisition. **Margaret J. Hosie:** Conceptualization, Formal analysis, Writing – original draft, Writing – review & editing, Supervision, Project administration, Funding acquisition.

## Declaration of Competing Interest

The authors declare that they have no known competing financial interests or personal relationships that could have appeared to influence the work reported in this paper.
